# Associations between cognitive impairment and computed tomography perfusion in different lobes in acute stroke of the anterior circulation

**DOI:** 10.1055/s-0043-1768663

**Published:** 2023-06-28

**Authors:** Mengna Chu, Bin Dong, Chao Huang

**Affiliations:** 1 Anhui Medical University, Second Affiliated Hospital, Department of Neurology, Hefei, China.; 2 Anhui Medical University, Third Affiliated Hospital, Department of Neurology, Hefei, China.

**Keywords:** Cognitive Dysfunction, Stroke, Cerebral Circulation, Disfunção Cognitiva, Acidente Vascular Cerebral, Circulação Cerebral

## Abstract

**Background** Cognitive impairment (CI) during the acute phase of stroke should not be ignored. The present study analyzed the relationship between computed tomography perfusion (CTP) in different lobes and CI during the acute phase of stroke in patients with cerebral infarction.

**Methods** The present study included 125 subjects: 96 in the acute phase of stroke and 29 elderly healthy subjects as a control group. The Montreal Cognitive Assessment (MoCA) was used to evaluate the cognitive status of the two groups. The CTP scans include four parameters: cerebral blood flow (CBF), cerebral blood volume (CBV), time to peak (TTP), and mean transit time (MTT).

**Results** The MoCA scores for naming, language and delayed recall significantly decreased only in patients with left cerebral infarctions. The MTT of the left vessels in the occipital lobe and the CBF of the right vessels in the frontal lobe were negatively related to the MoCA scores of patients with left infarction. The CBV of the left vessels in the frontal lobe and the CBF of left vessels in the parietal lobe were positively linked to the MoCA scores of patients with left infarction. The CBF of the right vessels in the temporal lobe was positively related to the MoCA scores of patients with right infarction. Finally, the CBF of the left vessels in the temporal lobe was inversely correlated with the MoCA scores of patients with right infarctions.

**Conclusion** During the acute phase of stroke, CTP was closely associated with CI. Changed CTP could be a potential neuroimaging biomarker to predict CI during the acute phase of stroke.

## Resumo

**Antecedentes** O comprometimento cognitivo (CC) durante a fase aguda do acidente vascular cerebral (AVC) não deve ser ignorado. O presente estudo analisou a relação entre perfusão por tomografia computadorizada (PTC) em diferentes lobos e CC durante a fase aguda do AVC em pacientes com infarto cerebral.

**Métodos** O presente estudo incluiu 125 indivíduos: 96 na fase aguda do AVC e 29 idosos saudáveis como grupo controle. O Montreal Cognitive Assessment (MoCA) foi usado para avaliar o estado cognitivo dos dois grupos. Os exames de PCT incluem quatro parâmetros: fluxo sanguíneo cerebral (FSC), volume sanguíneo cerebral (VSC), tempo até o pico (TAP) e tempo médio de trânsito (TMT).

**Resultados** Os escores do MoCA para nomeação, linguagem e recordação tardia diminuíram significativamente apenas em pacientes com infarto cerebral esquerdo. O TMT dos vasos esquerdos no lobo occipital e o FSC dos vasos direitos no lobo frontal foram negativamente relacionados aos escores MoCA de pacientes com infarto esquerdo. A VSC dos vasos esquerdos em o lobo frontal e o FSC dos vasos esquerdos no lobo parietal foram positivamente ligados aos escores MoCA de pacientes com infarto esquerdo. O FSC dos vasos direitos no lobo temporal foi positivamente relacionado com os escores MoCA de pacientes com infarto direito. Finalmente, o FSC dos vasos esquerdos no lobo temporal foi inversamente correlacionado com os escores MoCA de pacientes com infartos direitos.

**Conclusão** Durante a fase aguda do AVC, a PCT esteve intimamente associada ao CC. O PCT alterado pode ser um potencial biomarcador de neuroimagem para prever CC durante a fase aguda do AVC.

## INTRODUCTION

It has been demonstrated that cerebral infarction frequently results in cognitive dysfunction, also known as poststroke cognitive impairment (PSCI), which ranges from mild cognitive impairment (MCI) to dementia. Up to 4 years after a stroke, ∼ 23 to 30% of the patients with cerebral infarction develop MCI or dementia. 1 2 The 2016 American Heart Association/American Stroke Association (AHA/ASA) guidelines recommended that the cognitive status of stroke patients be assessed during hospitalization in the acute phase. 3 The first step toward reducing vascular risk factors and improving outcomes is to detect CI early in the acute phase of stroke. 4 5 As a result, CI during the acute phase should not be overlooked, and its potential risk factors and mechanism must be clinically evaluated.

Computed tomography perfusion (CTP) is a functional examination of brain tissue that characterizes the state of cerebral perfusion and has the highest accuracy in the diagnosis of anterior circulation ischemic stroke. 6 Furthermore, various studies 7 have recently revealed that CTP might become an important alternative for the visualization of cerebral and neuronal functional deficits in early dementia. There is a positive correlation between cognitive function and brain perfusion, and a study 8 has reported that significant brain perfusion corresponded to significant improvement in cognitive function. However, the relationship between CTP and CI during the acute phase of stroke remains unknown. Therefore, in the current study, we have analyzed the association between CTP in different lobes and CI during the acute phase of stroke in patients with cerebral infarction.

## METHODS

### Subjects

**Figure 1:**
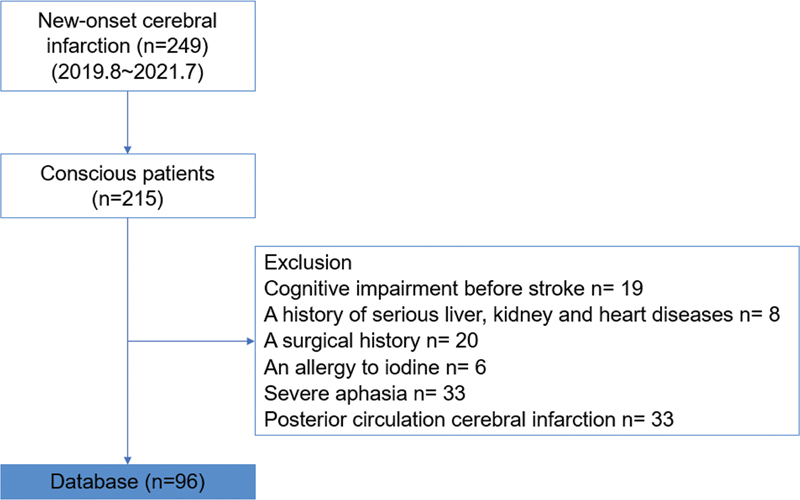
Flowchart of the recruitment and exclusion of patients.

We included 249 patients with new-onset cerebral infarction admitted to the Neurology Department of the Third Affiliated Hospital of Anhui Medical University between August 2019 and July 2021. After excluding several subjects who did not meet the inclusion criteria, 96 patients were included in the study ( Figure 1 ). The inclusion criteria were as follows: 1) patients who met the diagnostic criteria for cerebral infarction of the 2015 Cerebrovascular Disease Classification and were confirmed to have ischemic stroke by brain CT or diffusion-weighted imaging (DWI); 2) subjects whose first onset time was ≤ 72 hours; 3) patients aged between 45 and 79 years; 4) subjects with an etiology of acute cerebral infarction of the atherosclerotic type; 5) patients who were clearly conscious without severe aphasia (according to item 9 of the National Institutes of Health Stroke Scale [NIHSS]; the score of language plate is of ≤ 1 point); and 6) subjects with no recent history of surgery and trauma, with stable vital signs. The exclusion criteria were as follows: 1) subjects with CI, dementia, or other diseases that can affect cognitive and emotional functions before the onset of the disease; 2) patients with previous large-area cerebral infarction, cerebral hemorrhage, or traumatic brain injury; 3) subjects with a history of severe liver, kidney, and heart disease; 4) patients with a recent history of surgery and trauma, and those with unstable vital signs; 5) subjects who had taken drugs that affect cognitive function, mood and emotion in the previous month; 6) patients allergic to iodine who cannot undergo a contrast examination; and 7) subjects with severe visual or hearing impairment, dysarthria, aphasia, etc. who cannot cooperate with the examination. All subjects in the control group were aged between 45 and 79 years, without new infarctions on DWI, with a history of stroke, CI, dementia and mental illness, heart, lung, liver, and kidney dysfunction, and vision and hearing impairment, self-care, social adaptation is good, allergic to iodine, and their level of schooling was matched with that of the case group. The Ethics Committee of the Third Affiliated Hospital of Anhui Medical University (Heifei First Peoples Hospital) approved the study (under no. 2016[51])]. All participants provided written informed consent. All study procedures were conducted following the Declaration of Helsinki.

### Assessment of cognitive function

The Montreal Cognitive Assessment (MoCA) is a brief cognitive screening tool that has been used to assess cognitive function during hospitalization in the acute phase of an ischemic stroke (the first four weeks after onset). 5 9 10 11 Scores are assigned to seven domains of cognition: language, naming, attention, abstraction, delayed recall, visuospatial function, and orientation. 10 Cognitive function was classified as follows based on the recommended cutoff scores: 0 to 19–severe CI; 20 to 24–MCI; and 25 to 30–no CI. 12

### CTP imaging

A dual-source CT scanner was used to perform CTP after a conventional plain head CT scan (Somatom Definition Flash, Siemens Healthcare GmbH, Erlangen, Germany). The scanning parameters were as follows: tube voltage of 80 kV, tube current of 180 mA, slice thickness of 1.5 mm, pitch of 1.0 mm, collimation of 64 × 0.6 mm, and matrix of 512 × 512. The scanning was repeated 30 times at 1.5-second scan time and 4-second intervals. A double-tube high-pressure syringe was used to inject iohexol 350 mg/mL (50 mL) + saline (40 mL) intravenously into the right median cubital vein at 6 mL/s.

A computer was used to automatically eliminate vascular pixels to reduce the salience of the vascular structure. The CTP includes four parameters: cerebral blood flow (CBF), cerebral blood volume (CBV), time to peak (TTP), and mean transit time (MTT).

### Statistical analysis

The Statistical Package for the Social Sciences (SPSS for Widows, SPSS Inc., Chicago, IL, United States) software, version 16.0, was used for data analysis. The categorical data were expressed as numbers and percentages, and the continuous data, as mean and standard deviation (SD) values. The differences between the two groups were compared using the *t* -test; the rank-sum test was used to compare data with skewed distribution; the Chi-squared test was used to compare count data between the two groups; analysis of variance (ANOVA) was used to assess differences in measurement data among multiple groups; and the Duncan multi-range test was used. Statistical significance analysis was performed. A multivariate analysis was conducted using linear regression. Statistical significance was set at *p* < 0.05.

## RESULTS

### Baseline characteristics

At the baseline of observation analyses, 96 patients with new-onset anterior circulation cerebral infarction were included. Their mean age was of 63.93 (SD: ± 10.05) years, and 67 participants (69.79%) were men. The patients were divided into two groups based on the location of the lesion: left and right infarction groups. All baseline characteristics of the participants (96 patients and 29 controls) are displayed in Table 1 . Except for the homocysteine levels, no significant differences were found in terms of baseline characteristics among the participants.

**Table 1:** Baseline characteristics of the study participants

Variables		Controls ( *N* = 29)	Left infarction ( *N* = 38)	Right infarction ( *N* = 58)	*p*
Age (years): mean ± SD		65.28 ± 9.00	63.50 ± 9.93	64.21 ± 10.20	0.766
Sex: n (%)	Male	22 (75.9)	25 (65.8)	42 (72.4)	0.640
	Female	7 (24.1)	13 (34.2)	16 (27.6)	
Years of schooling		8.17 ± 3.40	7.03 ± 4.14	6.78 ± 4.26	0.307
Hypertension: n (%)	With	24 (82.8)	27 (71.1)	45 (77.6)	0.521
	Without	5 (17.2)	11 (28.9)	13 (22.4)	
Diabetes: n (%)	With	12 (41.4)	12 (31.6)	17 (29.3)	0.518
	Without	17 (58.6)	26 (68.4)	41 (70.7)	
Smoking status: n (%)	Never smoked	14 (48.3)	23 (60.5)	29(50.0)	0.514
	Current smoker	15 (51.7)	15 (39.5)	29 (50.5)	
Alcohol use: n (%)	Never	19 (65.5)	28 (73.7)	34 (58.6)	0.318
	Current user	10 (34.5)	10 (26.3)	24 (41.4)	
Carotid atherosclerosis: n (%)	With	25 (86.2)	37 (97.4)	54 (93.1)	0.214
	Without	4 (13.8)	1 (2.6)	4 (6.9)	
Atrial fibrillation: n (%)	With	1 (3.4)	2 (5.3)	2 (3.4)	0.893
	Without	28 (96.6)	36 (94.7)	56 (96.6)	
Cardiac function – ejection fraction: mean ± standard deviation		65.55 ± 5.30	66.24 ± 6.08	64.88 ± 5.43	0.511
Serum triglyceride level: mean ± SD		2.01 ± 2.10	2.16 ± 2.80	1.91 ± 1.83	0.965
Serum total cholesterol level: mean ± SD		4.13 ± 0.88	4.28 ± 1.20	4.50 ± 1.06	0.845
Serum high-density lipoprotein level: mean ± SD		1.13 ± 0.24	1.30 ± 0.74	1.14 ± 0.33	0.475
Serum low-density lipoprotein level: mean ± SD		2.19 ± 0.69	2.37 ± 0.84	2.49 ± 0.83	0.599
Homocysteine level: mean ± SD		8.78 ± 3.97	17.28 ± 14.96	16.68 ± 19.80	0.013
Uric acid level: mean ± SD		324.00 ± 96.00	320.10 ± 88.94	324.42 ± 94.26	0.981

Abbreviation: SD, standard deviation.

### Cognitive impairment at the acute stroke phase

**Figure 2:**
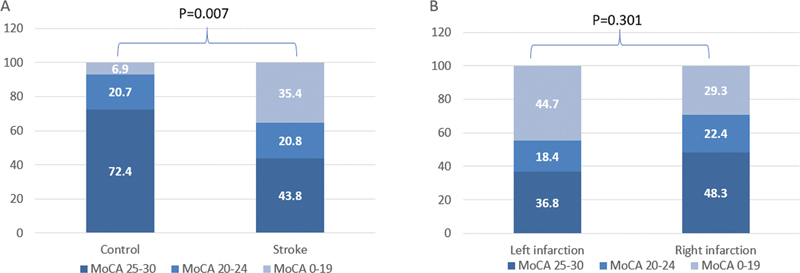
Cognitive impairment at the acute phase of stroke. ( **A** ) The patients with new-onset anterior circulation cerebral infarction experienced more cognitive impairment than the controls (56.3% versus 27.6% respectively; *p* = 0.007). ( **B** ) The cognitive impairment of the left infarction group did not differ from that of the right infarction group ( *p* = 0.301).

The patients with new-onset anterior circulation cerebral infarction had a higher rate of CI (MoCA score < 25) than the controls (56.3% versus 27.6% respectively; *p* = 0.007). The proportion of patients with MCI (MoCA score: 20 to 24) in the case group was comparable to that of the control group (20.8 versus 20.7% respectively), as depicted in Figure 2A . However, the proportion of patients with severe CI (MoCA score: 0 to 19) was higher (case group: 35.4%; controls: 6.9%). Furthermore, CI in the left infarction group was not different from that of the right infarction group ( Figure 2B ).

Moreover, no statistically significant differences in the score on the abstraction domain of the MoCA were found among the participants ( Table 2 ). In both the left and right infarction patients, the scores on the visuospatial/executive, attention, and orientation domains were significantly lower than those of the controls. Nevertheless, only those with left infarction scored significantly lower on the naming, language, and delayed recall domains.

**Table 2:** Participants' scores on the Montreal Cognitive Assessment (MoCA)

Domains	Controls ( *N* = 29)	Left infarction ( *N* = 38)	Right infarction ( *N* = 58)	*p*
Visuospatial/Executive: mean ± SD	3.93 ± 1.36	2.34 ± 1.98	2.78 ± 1.93	0.002
		*p* = 0.001	*p* = 0.012	
Naming: mean ± SD	2.86 ± 0.35	2.32 ± 0.99	2.55 ± 0.75	0.018
		*p* = 0.009	*p* = 0.133	
Attention: mean ± SD	6.34 ± 1.05	4.76 ± 2.07	5.43 ± 1.70	0.001
		*p* < 0.0001	*p* = 0.036	
Language: mean ± SD	2.97 ± 0.19	2.45 ± 0.83	2.74 ± 0.69	0.007
		*p* = 0.004	*p* = 0.228	
Abstraction: mean ± SD	1.07 ± 0.59	0.82 ± 0.77	0.90 ± 0.77	0.366
		*p* = 0.263	*p* = 0.461	
Delayed recall: mean ± SD	2.66 ± 1.45	1.74 ± 1.43	1.98 ± 1.50	0.037
		*p* = 0.023	*p* = 0.080	
Orientation: mean ± SD	5.52 ± 0.57	4.39 ± 1.67	4.71 ± 1.43	0.004
		*p* = 0.002	*p* = 0.019	

Abbreviation: SD, standard deviation.

### CTP parameters

The CBF, CBV, MTT, and TTP were measured in various areas of the brain, including the frontal, temporal, parietal, and occipital lobes, the basal ganglia, and the semioval center ( Table 3 ). Only MTT was significantly higher in the patients' frontal lobes than in those of the controls, while the CBF, CBV, and TTP remained unchanged.

**Table 3:** CTP parameters among the study participants

Parameters: mean ± (SD)		Controls ( *N* = 29)	Left infarction ( *N* = 38)	Right infarction ( *N* = 58)	*P*
**Frontal lobe**	CBF, right	55.84(± 5.97)	53.66(± 7.55)	53.98(± 9.07)	0.504
	CBF, left	57.23(± 6.23)	53.63(± 6.65)	55.18(± 7.33)	0.116
	CBV, right	3.26(± 0.29)	3.38(± 0.49)	3.48(± 0.74)	0.243
	CBV, left	3.28(± 0.30)	3.45(± 0.51)	3.52(± 0.90)	0.332
	MTT, right	4.01(± 0.76)	4.50(± 0.78)	4.59(± 0.96)	0.013
			*p* = 0.043	*p* = 0.007	
	MTT, left	3.86(± 0.63)	4.64(± 0.96)	4.46(± 0.92)	0.002
			*p* = 0.001	*p* = 0.008	
	TTP, right	10.51(± 2.03)	10.43(± 1.01)	10.54(± 1.42)	0.945
	TTP, left	10.67(± 1.03)	10.61(± 1.34)	10.07(± 1.38)	0.056
**Temporal lobe**	CBF, right	65.91(± 8.71)	65.87(± 9.91)	67.92(± 11.84)	0.572
	CBF, left	66.75(± 11.64)	58.81(± 13.63)	67.70(± 13.42)	0.004
			*p* = 0.030	*p* = 0.924	
	CBV, right	3.92(± 0.44)	4.11(± 0.59)	4.34(± 0.69)	0.012
			*p* = 0.351	*p* = 0.007	
	CBV, left	3.86(± 0.54)	4.03(± 0.77)	4.23(± 1.10)	0.189
	MTT, right	4.16(± 0.87)	4.40(± 0.69)	4.50(± 1.15)	0.304
	MTT, left	3.97(± 1.10)	5.01(± 1.58)	4.31(± 0.94)	0.002
			*p* = 0.001	*p* = 0.345	
	TTP, right	10.98(± 1.17)	10.35(± 0.99)	10.62(± 1.90)	0.258
	TTP, left	10.78(± 1.09)	11.32(± 2.27)	10.18(± 1.94)	0.017
			*p* = 0.400	*p* = 0.273	
**Parietal lobe**	CBF, right	57.10(± 8.28)	54.22(± 8.81)	51.32(± 11.15)	0.038
			*p* = 0.383	*p* = 0.022	
	CBF, left	58.50(± 8.57)	51.40(± 11.06)	52.63(± 10.98)	0.019
			*p* = 0.014	*p* = 0.030	
	CBV, right	3.39(± 0.34)	3.43(± 0.55)	3.44(± 0.77)	0.929
	CBV, left	3.47(± 0.36)	3.52(± 0.62)	5.30(± 10.46)	0.384
	MTT, right	4.21(± 0.74)	4.54(± 0.58)	4.94(± 1.21)	0.004
			*p* = 0.290	*p* = 0.003	
	MTT, left	4.24(± 0.75)	5.09(± 1.32)	4.68(± 1.02)	0.007
			*p* = 0.003	*p* = 0.128	
	TTP, right	11.31(± 1.06)	10.81(± 1.00)	11.26(± 1.56)	0.183
	TTP, left	11.22(± 1.08)	11.39(± 1.91)	10.66(± 1.44)	0.057
**Occipital lobe**	CBF, right	59.24(± 6.81)	59.88(± 11.95)	59.03(± 10.05)	0.920
	CBF, left	56.05(± 8.58)	52.93(± 14.31)	57.25(± 9.52)	0.174
	CBV, right	3.42(± 0.41)	3.75(± 0.74)	3.80(± 0.70)	0.044
			*p* = 0.088	*p* = 0.027	
	CBV, left	3.28(± 0.46)	4.84(± 7.53)	3.67(± 0.72)	0.264
	MTT, right	3.98(± 0.66)	4.47(± 0.77)	4.56(± 1.19)	0.032
			*p* = 0.074	*p* = 0.019	
	MTT, left	4.07(± 0.78)	5.15(± 1.73)	4.49(± 0.89)	0.001
			*p* = 0.001	*p* = 0.209	
	TTP, right	11.48(± 1.10)	10.89(± 1.02)	11.12(± 2.04)	0.330
	TTP, left	11.32(± 1.02)	11.50(± 2.12)	10.71(± 1.75)	0.074
**Semioval center**	CBF, right	47.99(± 5.59)	46.40(± 7.12)	44.73(± 8.14)	0.146
	CBF, left	48.68(± 5.10)	42.92(± 8.46)	44.82(± 7.88)	0.010
			*p* = 0.005	*p* = 0.050	
	CBV, right	2.91(± 0.30)	3.00(± 0.44)	3.04(± 0.52)	0.434
	CBV, left	2.93(± 0.29)	2.96(± 0.40)	2.99(± 0.82)	0.900
	MTT, right	4.27(± 0.69)	4.68(± 0.68)	5.02(± 1.16)	0.003
			*p* = 0.139	*p* = 0.001	
	MTT, left	4.24(± 0.68)	5.17(± 1.33)	4.86(± 1.16)	0.005
			*p* = 0.003	*p* = 0.033	
	TTP, right	11.39(± 1.09)	10.82(± 1.07)	11.34(± 1.60)	0.125
	TTP, left	11.21(± 1.06)	11.26(± 1.62)	10.77(± 1.42)	0.183
**Basal ganglia**	CBF, right	66.12(± 8.13)	63.25(± 8.03)	62.72(± 9.09)	0.216
	CBF, left	67.95(± 8.67)	60.90(± 13.90)	63.97(± 11.64)	0.061
			*p* = 0.033	*p* = 0.239	
	CBV, right	3.76(± 0.27)	3.82(± 0.46)	3.92(± 0.45)	0.222
	CBV, left	3.82(± 0.31)	3.95(± 0.69)	3.90(± 0.41)	0.577
	MTT, right	3.75(± 0.63)	4.04(± 0.59)	4.31(± 0.73)	0.002
			*p* = 0.152	*p* = 0.001	
	MTT, left	3.69(± 0.52)	4.53(± 1.27)	3.97(± 0.60)	0.000
			*p* = 0.000	*p* = 0.257	
	TTP, right	10.41(± 1.04)	9.84(± 1.26)	9.90(± 1.48)	0.180
	TTP, left	10.11(± 1.02)	10.09(± 1.45)	9.32(± 1.33)	0.005
			*p* = 0.998	*p* = 0.018	

Abbreviations: CBF, cerebral blood flow; CBV, cerebral blood volume; CTP, computed tomography perfusion; MTT, mean transit time; SD, standard deviation; TTP, time to peak.

The CBF, CBV, and MTT levels in the temporal lobe of the patients differed significantly from those of the controls. Surprisingly, significant differences in the CBF and MTT were found only in patients with left cerebral infarction. The CBF in the left vessels was considerably lower in these patients, while the MTT was significantly higher. However, the CBF and MTT in right infarction patients were comparable to those of the controls. The CBV of the right vessels increased significantly in right infarction patients; the CBV in the left infarction patients was similar to that of the controls.

Significant differences in terms of the CBF and MTT were found in the patients' parietal lobes compared with those of the controls. The CBF in both the left and right vessels was significantly lower in right infarction patients compared with the controls. In patients with left cerebral infarction, the CBF of the left vessels was significantly lower, and the MTT was significantly higher. The MTT of the right vessels also significantly increased in patients with right infarction.

Significant differences regarding the CBV and MTT were found in the occipital lobe of the patients compared with those of the controls. The MTT of the left vessels significantly increased in patients with left infarctions, and the MTT of the right vessels significantly increased in those with right infarctions. Furthermore, compared with the controls, the CBV of the right vessels increased significantly in right infarction patients, but remained unchanged in left infarction patients.

Moreover, in the comparison of the semioval center of patients and controls, there were significant differences in terms of CBF and MTT. The CBF of the left vessels was significantly reduced in patients with left cerebral infarctions. A significant increase was found in the MTT of the right vessels of right infarction patients. Similarly, the MTT of the left vessels increased significantly in both right and left infarction patients.

Finally, the CBF in the basal ganglia was significantly lower in patients with left cerebral infarction. On the other hand, the MTT levels in the left cerebral area of patients with left infarctions and in the right cerebral area of the patients with right infarction were significantly increased. In addition, the TTP of the right vessels was significantly higher in patients with right infarctions.

### Associations between cognitive impairment and CTP

During the linear regression analysis, data from left infarction patients were chosen to assess the relationship between CI and cerebrovascular reserve. This analysis revealed that the CBF of the right vessels in the frontal lobe, the CBV of the left vessels in the frontal lobe, CBF of the left vessels in the parietal lobe, and the MTT of the left vessels in the occipital lobes were risk factors and formed the regression equation ( *p* < 0.0001; Table 4 ). The CBF of the right vessels in the frontal lobe and the MTT of the left vessels in the occipital lobe were negatively related to the MoCA scores of patients with left infarction. The CBV of the left vessels in the frontal lobe and the CBF of the left vessels in the parietal lobe, on the other hand, were positively related to the MoCA scores of patients with left infarction.

**Table 4:** Linear regression analysis for cognitive impairment against CTP parameters

Factor		B	S.E.	*p*
**Left infarction**	Constant	27.746	8.382	0.002
	CBF, right, frontal lobe	-0.600	0.153	0.000
	CBV, left, frontal lobe	5.046	2.121	0.023
	CBF, left, parietal lobe	0.255	0.089	0.007
	MTT, left, occipital lobe	-1.215	0.554	0.035
**Right infarction**	Constant	22.750	6.082	0.000
	CBF, left, temporal lobe	-0.300	0.104	0.006
	CBF, right, temporal lobe	0.202	0.091	0.030

Abbreviations: B, beta, regression coefficient; S.E., standard error; CBF, cerebral blood flow; CBV, cerebral blood volume; CTP, computed tomography perfusion; MTT, mean transit time; TTP, time to peak.

A linear regression analysis was also performed regarding the data from right infarction patients to assess the relationship between CI and CTP. The CBF in the temporal lobe was a risk factor and was included in the regression equation ( *p* = 0.010, Table 4 ). The CBF of the right vessels in the temporal lobe was positively related to the MoCA scores of patients with right infarction. However, the CBF of the left vessels in the temporal lobe was negatively associated with the MoCA scores of these patients.

## DISCUSSION

Cognitive function has been reported to be a predictor of the development of PSCI in the acute stroke setting. 5 13 In the study by Lee et al. 14 (2020), 63.8% of the patients with acute ischemic stroke had CI (28.8% had MCI and 35.0% had severe CI). As a result, patients in the acute phase may experience a general decline in cognitive efficiency. In the present study, we found that 56.3% (20.8% with MCI and 35.4% with severe CI) of the patients with new-onset anterior circulation cerebral infarction had CI at the acute phase of stroke, which is consistent with previous studies.

The location of the lesion also affects the PSCI. 15 A study 16 with a large cohort of 410 acute ischemic stroke patients revealed that the crucial structures for PSCI are in the left anterior circulation, such as the angular gyrus and the basal ganglia. Furthermore, Weaver et al. 17 (2021) analyzed data from 2,950 acute ischemic stroke patients and concluded that the appearance of infarction in the left frontal lobe, left temporal lobe, left thalamus, and right parietal lobe was closely related to PSCI. According to Ni et al. 18 (2021), an asymmetry in hemodynamics makes the left hemisphere more vulnerable to various risk factors than the right hemisphere. In the present study, 63.1% of the patients with left infarction and 51.7% of those with right infarction presented with CI. The proportion of CI was only slightly higher in the left infarction group than in the right infarction group. This could be due to the small sample size, and, with larger samples, this difference may become more pronounced.

The MTT may be a more accurate marker of perfusion changes relative to other time-based perfusion parameters. 19 The present study indicated that the MTT of the patients was significantly increased compared with that of the controls in all areas of the brain (frontal, temporal, parietal, and occipital lobes, basal ganglia, and semioval center). Furthermore, the MTT increase in the ipsilateral side of the lesion was greater. In the study by Chen et al. 20 (2006), MTT increases were more likely to appear in the areas with severely-reduced perfusion reserves than in those with moderately-reduced or normal perfusion reserves. As for the CBF parameter, it is reduced when a ischemic stroke occurs and the CBF is inadequate. 21 The CBF was significantly lower in the temporal and parietal lobes, semioval center, and basal ganglia of the patients of the present study, and it was also reduced significantly on the ipsilateral side of the lesion. In addition, we found that the CBV was significantly higher in the right occipital lobe of right cerebral infarction patients. According to Ogasawara et al. 22 (2002), an increase in the CBV can be an early indicator of cerebral perfusion pressure. Overall, these results demonstrated that the CTP decreased in patients with new-onset anterior circulation cerebral infarction and was associated with the lesion. The present study also revealed that the MoCA scores of left infarction patients were negatively related to the CBF in the right frontal lobe and the MTT in the left occipital lobe while positively related to the CBV in the left frontal lobe and the CBF in the left parietal lobe.

Moreover, the MoCA scores of patients with right infarction were negatively associated with the CBF in the left temporal lobe. Concurrently, a positive association was found with the CBF in the right temporal lobe. These findings suggest that regional CBF may be a more sensitive CI indicator, which is in line with previous studies. Yin et al. 23 (2019) reported that the CBF could be used as a candidate imaging indicator to monitor global cognitive function changes in patients with cerebral autosomal dominant arteriopathy with subcortical infarcts and leukoencephalopathy.

In conclusion, CI was associated with CTP in new-onset anterior circulation cerebral infarction patients, particularly during the acute phase of the stroke. Finally, changed CTP could be a potential neuroimaging biomarker to predict CI during the acute phase of stroke.
